# Budesonide repairs decreased barrier integrity of eosinophilic nasal polyp epithelial cells caused by PM_2.5_

**DOI:** 10.1002/clt2.12029

**Published:** 2021-07-03

**Authors:** Siyuan Ma, Mu Xian, Yang Wang, Chengshuo Wang, Luo Zhang

**Affiliations:** ^1^ Department of Otolaryngology Head and Neck Surgery Beijing TongRen Hospital Capital Medical University Beijing China; ^2^ Beijing Key Laboratory of Nasal Diseases Beijing Institute of Otolaryngology Beijing China; ^3^ Research Unit of Diagnosis and Treatment of Chronic Nasal Diseases Chinese Academy of Medical Sciences Beijing China; ^4^ Department of Allergy Beijing TongRen Hospital Capital Medical University Beijing China

**Keywords:** budesonide, epithelial cells, nasal barrier, particular matter 2.5, type‐2 inflammation

## Abstract

**Background:**

Eosinophilic chronic rhinitis with nasal polyps (eos‐CRSwNP) is a subtype of nasal polyps (NPs) characterized by severe type‐2 inflammation and defective epithelial barrier function. The epithelial barrier plays important roles in the pathogenesis of NPs and type‐2 inflammation. Particular matter 2.5 (PM_2.5_) are fine particles with a diameter less than 2.5 μm, containing a mixture of different components. Here, we investigated the impact of PM_2.5_ on the barrier function of the eos‐CRSwNP epithelium and explored the reparative function of budesonide.

**Methods:**

Samples from noninflammatory nasal mucosa and eos‐CRSwNP were collected to establish an in vitro air–liquid interface cultured model. The cells were exposed to PM_2.5_ at 50 or 100 µg/ml intermittently for 72 h, with or without budesonide pretreatment. Barrier function and tight junction (TJ) expression were reflected by measuring transepithelial resistance (TER), paracellular flux permeability of fluorescein isothiocyanate‐labeled 4‐kDa dextran, quantitative real‐time polymerase chain reaction (qPCR), and immunofluorescence staining of TJ proteins. Cytokine expression was measured by qPCR and enzyme‐linked immunosorbent assay or Luminex.

**Results:**

PM_2.5_ increased paracellular flux and downregulated TJ protein expression (zona occuldens‐1, occludin, and claudin‐1), but did not change TER. These changes could be partially restored by budesonide treatment. Interleukin (IL)‐8, IL‐10, IL‐1*α*, and tissue inhibitor of metalloproteinase (TIMP)‐1 concentrations were significantly increased in the culture medium of cells exposed to PM_2.5_, and budesonide significantly reduced the changes in IL‐8, IL‐1*α*, and TIMP‐1.

**Conclusion:**

PM_2.5_ impaired the barrier function of eos‐CRSwNP epithelial cells and increased the permeability of large molecules. PM_2.5_ also increased the secretion of pro‐inflammatory cytokines by nasal epithelial cells. Budesonide could partially repair the damage, suggesting potential applications in clinical practice.

## 1 INTRODUCTION

According to the World Health Organization,^1^ air pollution is a major health threat worldwide. Particular matter 2.5 (PM_2.5_) are fine particles containing a range of different components, including acids (such as nitrate and sulfates), organics, metals, and soil or dust particles.^2^ Many studies have demonstrated that PM_2.5_ is closely related to human diseases and mortality, causing systematic inflammation and affect the immune system.^2012^, ^2018^

As the first line of immunological defense, nasal epithelial cells provide a physical barrier to prevent the invasion of inhaled environmental pathogens, allergens, and other airborne irritants.^5^ Apical junctional complexes, which consist of apical tight junctions (TJs) and underlying adherens junctions (AJs), are the most important part of the epithelial barrier.

The prevalence of the type‐2 nasal inflammatory disease is rising rapidly. Previous studies have shown that dysregulation of the epithelial cell response or barrier function could drive chronic type‐2 inflammation,^6^ including many nasal inflammatory diseases.^7^ Eosinophilic chronic rhinitis with nasal polyps (eos‐CRSwNP) is a type‐2 inflammatory disease characterized by a tendency for recurrence after surgery^8^ and usually shows sensitivity to corticosteroid treatment.^9^ Eos‐CRSwNP patients are also more likely to have other allergic comorbidities and higher blood eosinophilia percentage.^10^

Previous studies considered that PM_10_ have more effect on the upper airway, recent studies began to raise attention on the effects of PM_2.5_. One recent epidemiological study in Europe^11^ confirmed that among all the factors they assessed about air quality (PM_10_, PM_2.5_, PM_corase_, NO_2_, traffic load, and traffic intensity), PM_2.5_ and PM_10_ were the only two showed a clear relationship with severity of symptoms in the upper airway. Our previous study using noninflammatory human nasal epithelial cells demonstrated that PM_2.5_ causes deficiencies in barrier integrity, whereas steroids fail to repair this damage.^12^ However, whether PM_2.5_ can exacerbate damage to inflammatory cells is still unclear.

Accordingly, in this study, we evaluated the impact of PM_2.5_ on the air–liquid interface (ALI) of cultured eos‐CRSwNP epithelial cells and further explored the underlying mechanisms and reparative effects of steroids.

## 2 MATERIALS AND METHODS

### 2.1 Patients

Patients who underwent nasal endoscopic surgery at Beijing Tongren Hospital from May to August 2018 were randomly enrolled in the study. The criteria of eos‐CRSwNP subjects are listed as follows: (1) adults (18–70 years old) from Beijing or other cities in north China; (2) patients with endoscopically visible nasal polyp (NP); (3) patients with a history of smoking, taking oral/intranasal steroid, or systematic disease that might influence the results of this study were excluded; (4) all of the NP samples underwent histopathological inspection, and those with tissue eosinophils percentage less than 27% were excluded from this study. The nasal uncinate processes from patients undergoing surgery for septal deformities, cerebrospinal fluid leak, or bullous middle turbinate were collected as noninflammatory nasal mucous tissues. The diagnosis of CRSwNP was consistent with EPOS 2012,^13^ and the NPs were classified as eosinophilic when the percentage of tissue eosinophils exceeded 27% of all infiltrated inflammatory cells.^10^ Patients with a history of smoking and taking oral/intranasal steroid medicine within 3 weeks were excluded from this study. Twelve patients with eos‐CRSwNP and five patients with noninflammatory nasal disease were included in this study; clinical data for these patients are shown in Table 1.

**TABLE 1:** Patient clinical data

Operation date	Gender	Age	Diagnose	Blood eosinophil percentage	Hyposmia	Allergic rhinitis	Asthma	Dermatitis	Drug allergy	NERD	Allergen sensitization profile	Other diseases
14‐Jun‐18	Female	41	eos‐CRSwNP	2.4	Yes	No	No	No	Aspirin	Yes	Negative	None
28‐Jun‐18	Male	40	eos‐CRSwNP	11	Yes	Yes	Yes	Yes	N	No	Negative	Renal cyst
29‐Jun‐18	Male	49	eos‐CRSwNP	3.3	Yes	No	No	No	N	No	House dust mites	None
29‐Jun‐18	Female	56	eos‐CRSwNP	3.1	Yes	No	No	No	N	No	Negative	None
20‐Jul‐18	Male	48	eos‐CRSwNP	23.9	Yes	Yes	Yes	Yes	N	No	Negative	None
01‐Aug‐18	Male	54	eos‐CRSwNP	12	Yes	Yes	Yes	Yes	N	No	Negative	Diabetes
02‐Aug‐18	Male	46	eos‐CRSwNP	9.2	Yes	No	No	No	N	No	Negative	None
22‐May‐18	Male	51	eos‐CRSwNP	0.8	Yes	No	No	No	N	No	Negative	Hypertension
22‐May‐18	Male	36	eos‐CRSwNP	3.8	Yes	Yes	No	Yes	N	No	Negative	None
07‐Jun‐18	Male	63	eos‐CRSwNP	1.8	Yes	Yes	No	No	N	No	Negative	None
07‐Jun‐18	Male	58	eos‐CRSwNP	6.8	Yes	No	No	No	N	No	Negative	None
07‐Jun‐18	Female	70	eos‐CRSwNP	15.5	Yes	Yes	Yes	Yes	Ibuprofen	Yes	Negative	Hypertension
13‐Jul‐18	Female	50	Septal deviation	0.11	No	No	No	No	No	No	Negative	None
29‐Aug‐18	Male	26	Septal deviation	0.78	No	No	No	Yes	No	No	Negative	None
14‐Jul‐18	Female	46	Septal deviation	0.17	No	No	No	No	No	No	Negative	None
19‐Jun‐18	Female	51	Cerebrospinal fluid rhinorrhea	0.13	No	No	No	No	No	No	Negative	None
09‐Jul‐18	Male	24	Septal deviation	0.4	No	No	No	No	No	No	Negative	None

Abbreviations: eos‐CRSwNPn, eosinophilic chronic rhinitis with nasal polyps; NERD, NSAIDs‐exacerbated respiratory disease.

### 2.2 Human nasal epithelial cells culture

Fresh noninflammatory nasal mucous tissues and NP samples were collected during endoscopic surgery. After isolation, the samples were washed with phosphate‐buffered saline (PBS) with 2× penicillin‐streptomycin (200 U/ml penicillin, 200 μg/ml streptomycin; Thermo Fisher Scientific). After incubation in Dulbecco's modified Eagle's medium (Lonza) with 2× penicillin‐streptomycin for 2 h in 4°C, the samples were washed again with PBS, digested, seeded, and cultured as described in our previous study.^12^

### 2.3 Preparation and cytotoxicity of PM_2.5_

PM_2.5_ was a gift from the Institute for Environmental Health and Related Product Safety, Chinese Center for Disease Control and Prevention. The purification procedure, chemical composition, and methods to measure the cytotoxicity of PM_2.5_ in eos‐CRSwNP epithelial cells were described in our previous study.^12^

### 2.4 Transepithelial electrical resistance and paracellular flux measurement in ALI‐cultured cells

After 21 days of ALI culture for cell differentiation, when the transepithelial electrical resistance (TER) reached a plateau of more than 300 Ω*cm,^2^ the cultures were ready to be used for further experiments. The TER was measured using a Millicell‐ERS Volt Ohm Meter (Millipore). Similar to our previous study, the cultures were exposed to 50 or 100 μg/ml PM_2.5_ or culture medium as a control for 8 h each day in the apical chamber. The cells were then washed three times with PBS. To investigate the effects of budesonide, 1 μM budesonide (Sigma‐Aldrich) was added to the apical chamber at 1 h before PM_2.5_ exposure.

TER was measured at 0, 24, 48, and 72 h in triplicate after initiation of exposure. After 72‐h TER measurement, the paracellular permeability of the cell layer was evaluated by adding 2 mg/ml fluorescein isothiocyanate (FITC)‐labeled 4‐kDa dextran (Sigma‐Aldrich) to the apical layer of the ALI cells. After 12 h, the concentration of FITC in the basolateral medium was measured using an enzyme‐linked immunosorbent assay (ELISA) reader (Mithra LB 940; Berthold Technologies) at 480 nm in duplicate.

### 2.5 RNA isolation and quantitative real‐time polymerase chain reaction

At 72 h after PM_2.5_ exposure, total RNA was extracted and purified with a MiniBEST Universal RNA Extraction Kit (TaKaRa Biotechnology), according to the manufacturer's instructions. A NanoDrop 2000 Spectrophotometer (Thermo Fisher Scientific) was used to measure the quantity and quality of the isolated RNA.

PrimeScript RT Master Mix (TaKaRa Biotechnology) was used to synthesize single‐stranded cDNA, and quantitative real‐time polymerase chain reaction (qPCR) was performed using a SYBR Premix Ex Taq kit (TaKaRa Biotechnology) on an Applied Biosystems ViiA 7 Dx System (Applied Biosystems). Details of the primers used in this study are described in Table S1.

### 2.6 Immunofluorescence staining

At 72 h after PM_2.5_ exposure, ALI cultures were fixed in a 1:1 mixture of methanol‐acetone for 10 min at 4°C. The cells were then washed in PBS and blocked with 5% skim milk. The methods for staining, examining, and analyzing were the same as described in our previous study.^12^

### 2.7 Luminex and ELISA analyses

At 72 h after PM_2.5_ exposure, the culture medium in the basolateral chamber was collected and stored in −80°C. Samples were tested using a Human Magnetic Luminex Screening Assay (R&D), which included interleukin (IL)‐*α*, IL‐10, tissue inhibitor of metalloproteinase (TIMP)‐1, IL‐25, and IL‐33. IL‐8 and thymic stromal lymphopoietin (TSLP) concentrations in samples were also examined using ELISA kits (R&D and ThermoFisher Scientific, respectively). Total protein concentrations were determined using BCA Protein Assay Kits (Beyotime). The tests were performed according to the manufacturer's recommendations, and Luminex Assays and Protein Assays were evaluated on a Bio‐Plex 200 System (Bio‐Rad Laboratories) and ELISA reader (Mithra LB 940), respectively.

### 2.8 Statistical analysis

All data were analyzed using GraphPad Prism 8 software (GraphPad Software). Data are presented as median and interquartile range unless otherwise noted. Wilcoxon matched‐pairs signed‐rank tests were used to analyze differences between two paired groups, and Mann–Whitney U‐tests were used to analyze two unmatched groups. For the comparison of multiple groups, Friedman test was used, and posttest was performed to analyze the results from matched groups. Results with *p* values of less than 0.05 were considered statistically significant.

## 3 RESULTS

### 3.1 PM_2.5_ caused impairment of barrier integrity in eos‐CRSwNP epithelial cells

Compared with that of noninflammatory nasal epithelial cells, the TER baseline of eos‐CRSwNP epithelial cells was lower after 21 days of ALI culture (Figure S1A). Additionally, the FITC‐labeled 4‐kDa dextran concentration in the culture medium of eos‐CRSwNP epithelial cells tended to be higher than that in the culture medium of noninflammatory nasal epithelial cells (Figure S2). Similar to its toxicity in normal nasal epithelial cells, concentrations of 50 and 100 μg/ml PM_2.5_ resulted in the death of less than 10% eos‐CRSwNP epithelial cells (Figure S3); therefore, these concentrations were used for treatment of well‐differentiated ALI cultures.

**FIGURE 1:**
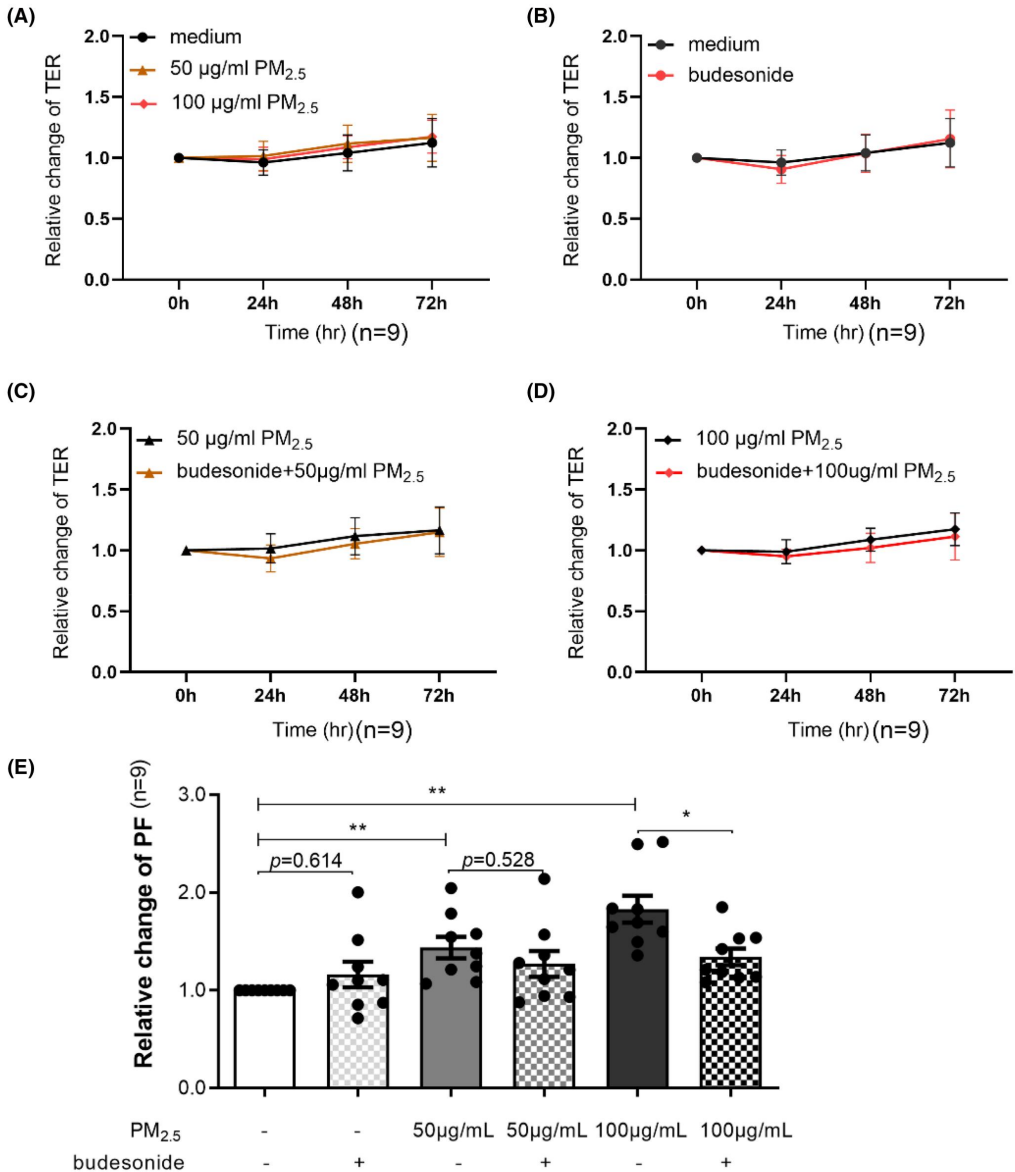
Effects of PM_2.5_ on TER and paracellular flux. (A) Effects of 50 and 100 μg/ml PM_2.5_ on TER after 24, 48, and 72 h of intermittent exposure compared with the cell culture medium control. (B–D) Effects of budesonide pretreatment on TER 1 h before exposure to PM_2.5_. (E) Effects of PM_2.5_ and budesonide on FITC‐dextran paracellular flux in ALI‐cultured eos‐CRSwNP epithelial cells after 72 h of exposure.*p < 0.05, **p < 0.01. ALI, air–liquid interface; eos‐CRSwNP, eosinophilic chronic rhinitis with nasal polyps; FITC, fluorescein isothiocyanate; PM_2.5_, particular matter 2.5; TER, transepithelial resistance

In the continuous monitoring of TER, the relative TER (ratio at each time point of TER to that at 0 h) did not change significantly (Figure 1A) in each group. However, at 72 h after exposure, the relative paracellular flux, which was expressed as a ratio of FITC‐labeled 4‐kDa dextran concentration in each group to that of culture medium group, showed a significant, concentration‐dependent increase (50 μg/ml PM_2.5_: 1.379 [1.149, 1.682]; *p* < 0.01; 100 μg/ml PM_2.5_:1.693 [1.549, 2.167]; *p* < 0.01; Figure 1E).

### 3.2 Impairment of barrier integrity caused by PM_2.5_ could be partially repaired by budesonide

Although budesonide pretreatment did not influence the relative TER (Figure 1B–D), it partially reversed the increase in relative paracellular flux caused by PM_2.5_. After intermittent exposure to 100 μg/ml PM_2.5_ for 72 h, the relative paracellular flux showed a significant difference between the groups with or without budesonide pretreatment (with budesonide vs. without budesonide:  1.210 [1.134, 1.534] vs. 1.693 [1.549, 2.167]; *p* < 0.05; Figure 1E).

### 3.3 Changes in barrier integrity caused by PM_2.5_ were different between noninflammatory nasal epithelial cells and eos‐CRSwNP epithelial cells

After exposure to 100 μg/mL PM_2.5_ for 72 h, the TER of eos‐CRSwNP epithelial cells tended to increase slightly, whereas that in noninflammatory nasal epithelial cells decreased significantly compared with that in the eos‐CRSwNP group (noninflammatory vs. eos‐CRSwNP: 0.815 [0.404, 0.952] vs. 1.159 [0.863, 1.418], *p* < 0.01; Figure S1B). However, after 72 h of PM_2.5_ exposure, the paracellular flux increased in both types of cells (noninflammatory: 0.147 [0.134, 0.194] vs. 0.545 [0.348, 0.843] mg/ml; eos‐CRSwNP: 0.355 [0.264, 0.404] vs. 0.644 [0.527, 0.670] mg/ml; *p* < 0.01; Figure S2).

Following budesonide pretreatment, the changes in TER caused by 100 μg/ml PM_2.5_ showed much more difference between those two cell types (Figure S1C,D). Because the paracellular flux of eos‐CRSwNP epithelial cells could be restored by budesonide, FITC‐dextran 4 kDa in the basolateral medium of eos‐CRSwNP was significantly lower than noninflammatory epithelium cells after been pretreated by 1 μmol/L budesonide (noninflammatory vs. eos‐CRSwNP: 0.741 [0.632, 0.870] vs. 0.452 [0.380, 0.503], *p* < 0.01; Figure S2).

### 3.4 Effects of PM_2.5_ on the expression of TJ‐related mRNAs and proteins in eos‐CRSwNP epithelial cells

**FIGURE 2:**
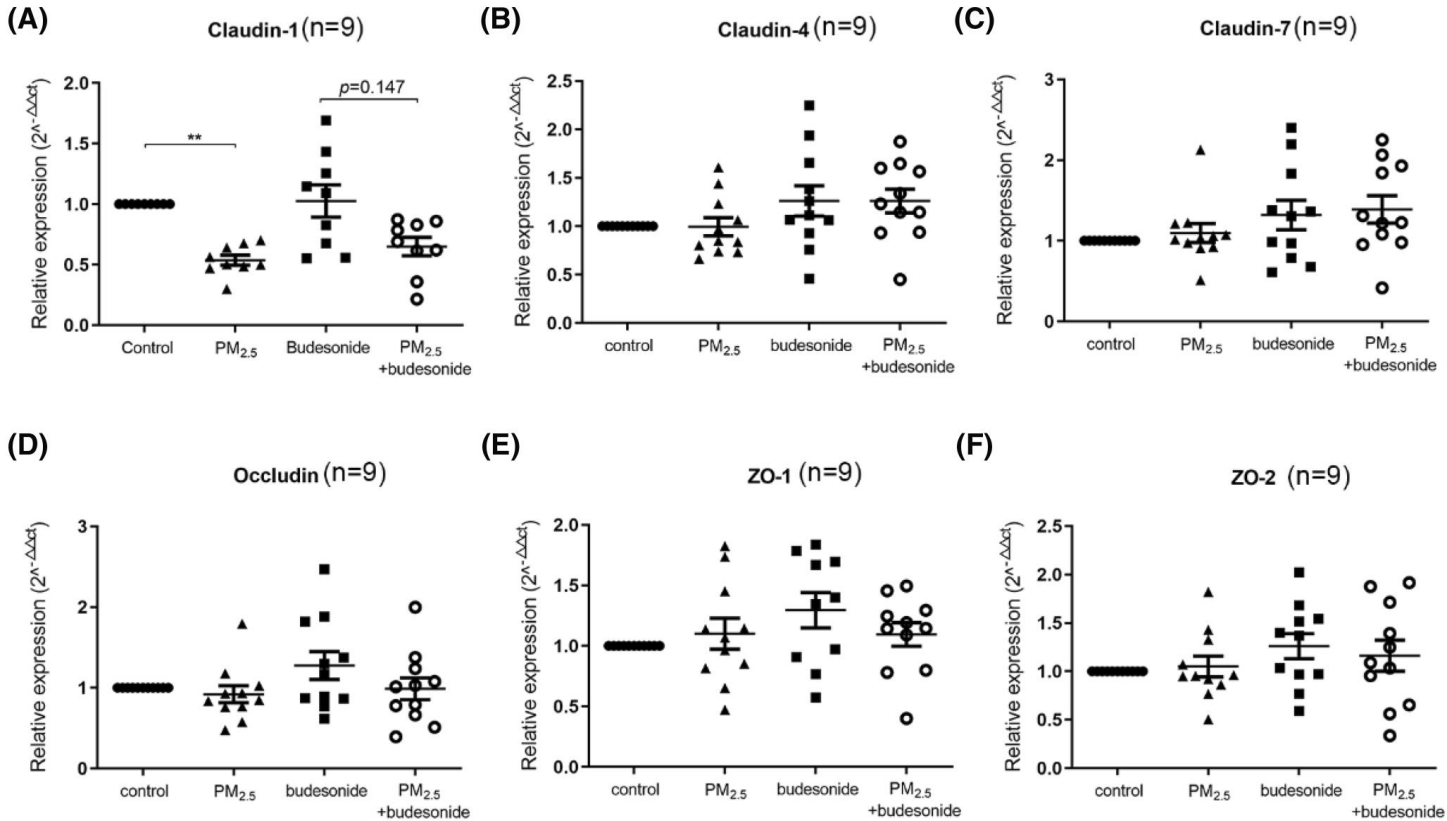
Effects of PM_2.5_ on the mRNA expression of TJ‐related proteins. qPCR was used to evaluate changes in claudin‐1, claudin‐4, claudin‐7, occludin, ZO‐1, and ZO‐2 after intermittent exposure to 100 μg/ml PM_2.5_ for 72 h, with or without budesonide pretreatment. **p < 0.01. qPCR, quantitative real‐time polymerase chain reaction; PM_2.5_, particular matter 2.5; TJ, tight junction; ZO, zona occludens

In eos‐CRSwNP epithelial cells exposed to 100 μg/ml PM_2.5_, the expression of claudin‐1 was significantly decreased (1.0 [1.0, 1.0] vs. 0.502 [0.478, 0.659], *p* < 0.01), whereas that of claudin‐4, claudin‐7, occludin, zona occludens (ZO)‐1, and ZO‐2 did not change significantly. However, when the cells were pretreated with budesonide for 1 h prior to PM_2.5_ exposure, the expression of claudin‐1 was still significantly decreased, but tended to recover. The expression levels of other TJ‐related mRNAs tended to increase, but did not change significantly in the budesonide group compared with those in the nonpretreated group (Figure 2).

**FIGURE 3:**
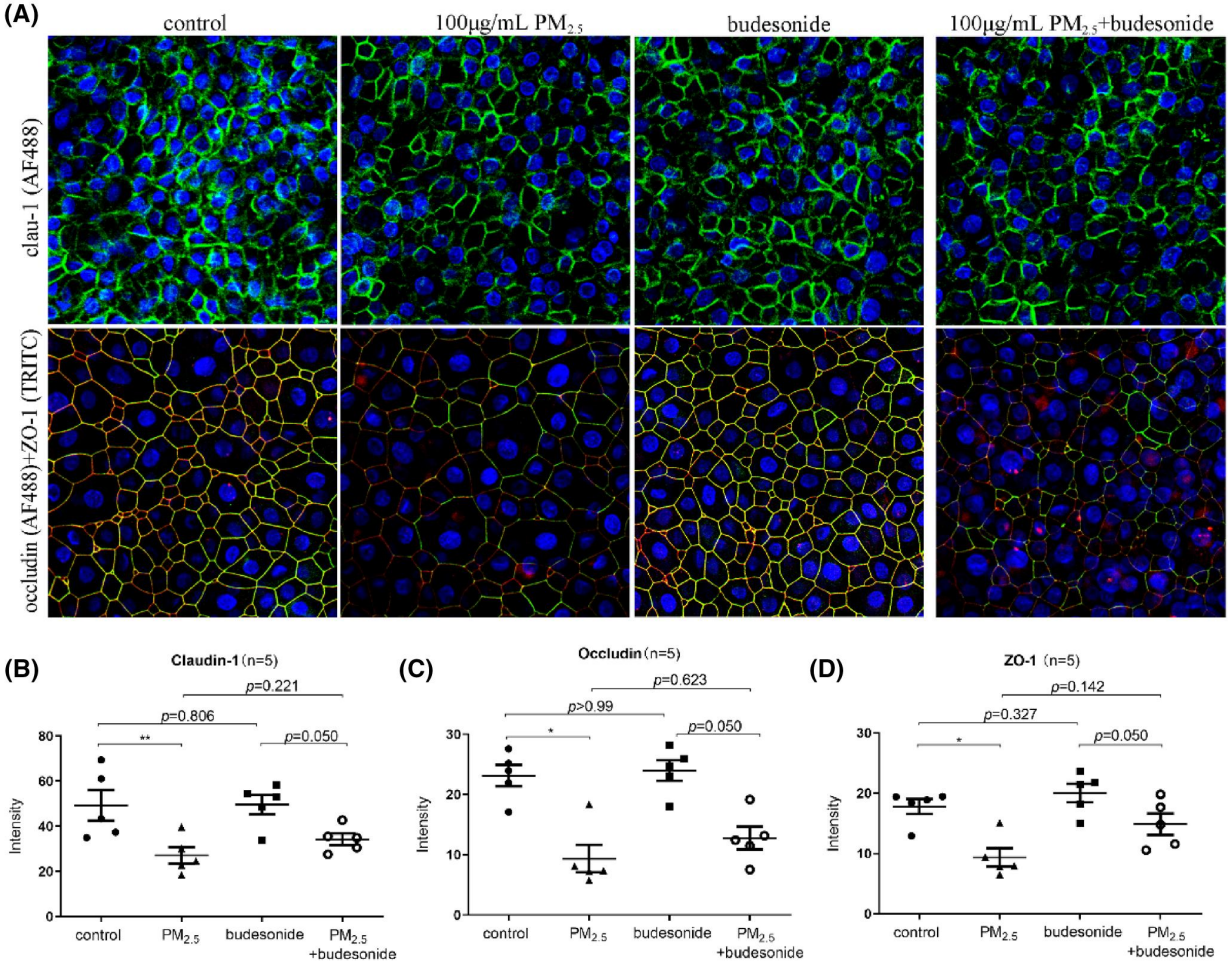
Expression of TJ‐related proteins exposed to PM_2.5_ and budesonide. (A) Representative images of immunofluorescence staining for claudin‐1, occludin, and ZO‐1 in ALI‐cultured eos‐CRSwNP epithelial cells (400× magnification). (B) Fluorescence intensities were evaluated using ImageJ software. *p < 0.05, **p < 0.01. ALI, air–liquid interface; eos‐CRSwNP, eosinophilic chronic rhinitis with nasal polyps; PM_2.5_, particular matter 2.5; TJ, tight junction; ZO, zona occludens

The expression levels of claudin‐1, ZO‐1, and occludin were then evaluated by confocal fluorescence microscopy and semiquantified using ImageJ software. After exposure to PM_2.5_, the signals for TJ‐related proteins were weaker and ruptured. The budesonide pretreatment partially enhanced the expression of TJ‐related proteins (Figure 3).

### 3.5 Effects of PM_2.5_ on cytokine secretion by eos‐CRSwNP epithelial cells

**FIGURE 4:**
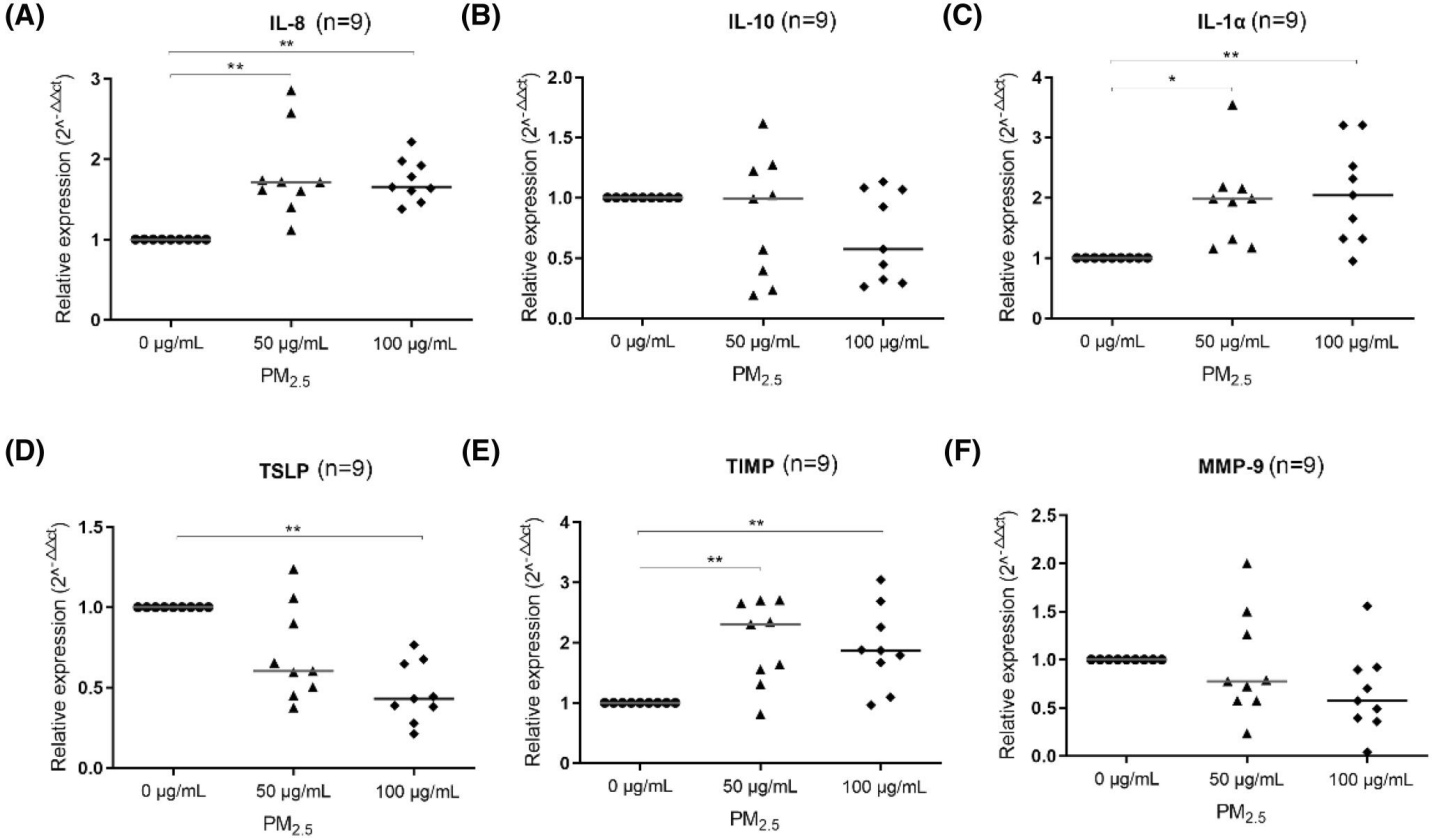
Effects of PM_2.5_ on cytokine mRNA expression. After intermittent exposure to 50 or 100 μg/ml PM_2.5_ for 72 h, levels of IL‐8, IL‐1*α*, TIMP, TSLP, IL‐10, and MMP‐9 mRNA in cultured cells were evaluated. *p < 0.05, **p < 0.01. IL, interleukin; MMP, matrix metalloproteinase; PM_2.5_, particular matter 2.5; TIMP, tissue inhibitor of metalloproteinase; TSLP, thymic stromal lymphopoietin

At the mRNA level, the expression levels of IL‐8, IL‐1*α*, and TIMP were significantly increased in both the 50 and 100 μg/ml PM_2.5_ groups. Moreover, IL‐1*α* was upregulated in a concentration‐dependent manner. The expression of TSLP mRNA was significantly decreased in the 100 μg/ml PM_2.5_ group, and IL‐10 and matrix metalloproteinase‐9 (MMP‐9) tended to decrease, but the difference was not significant (Figure 4).

**FIGURE 5:**
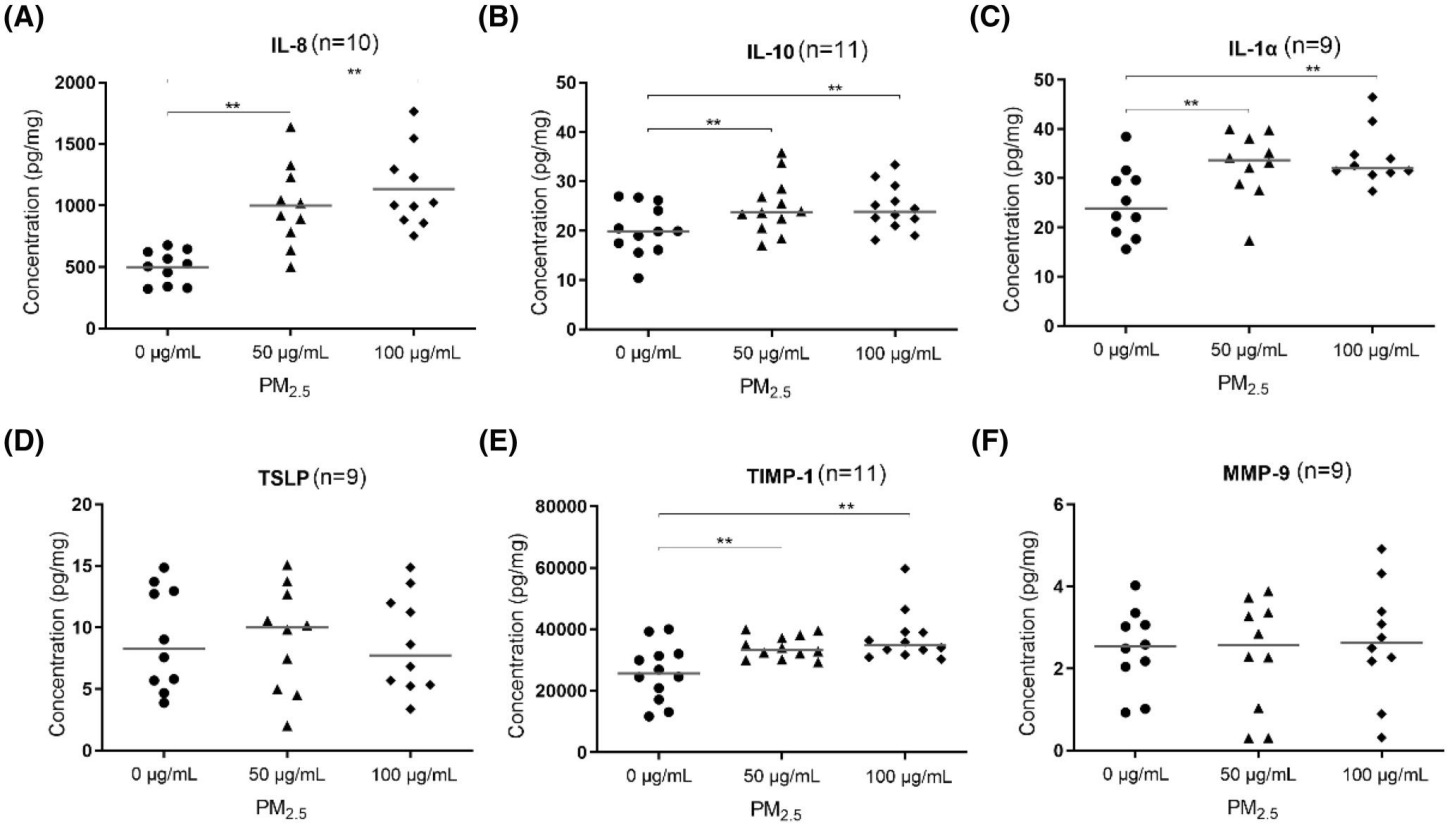
Cytokine concentrations in the culture medium after exposure to PM_2.5_. After intermittent treatment with 50 or 100 μg/ml PM_2.5_ for 72 h, levels of IL‐8, IL‐10, IL‐1*α*, TIMP‐1, TSLP, and MMP‐9 in the culture medium were evaluated. *p < 0.05, **p < 0.01. IL, interleukin; MMP, matrix metalloproteinase; PM_2.5_, particular matter 2.5; TIMP, tissue inhibitor of metalloproteinase; TSLP, thymic stromal lymphopoietin

The detected concentrations of IL‐25 and IL‐33 were lower than the limit of detection for the Luminex kits used. The results of ELISA and Luminex analyses showed that in the cell culture medium, the concentrations of IL‐8, IL‐10, IL‐1*α*, and TIMP‐1 increased significantly in both the 50 and 100 μg/ml PM_2.5_ groups. However, TSLP and MMP‐9 expression levels did not change (Figure 5).

**FIGURE 6:**
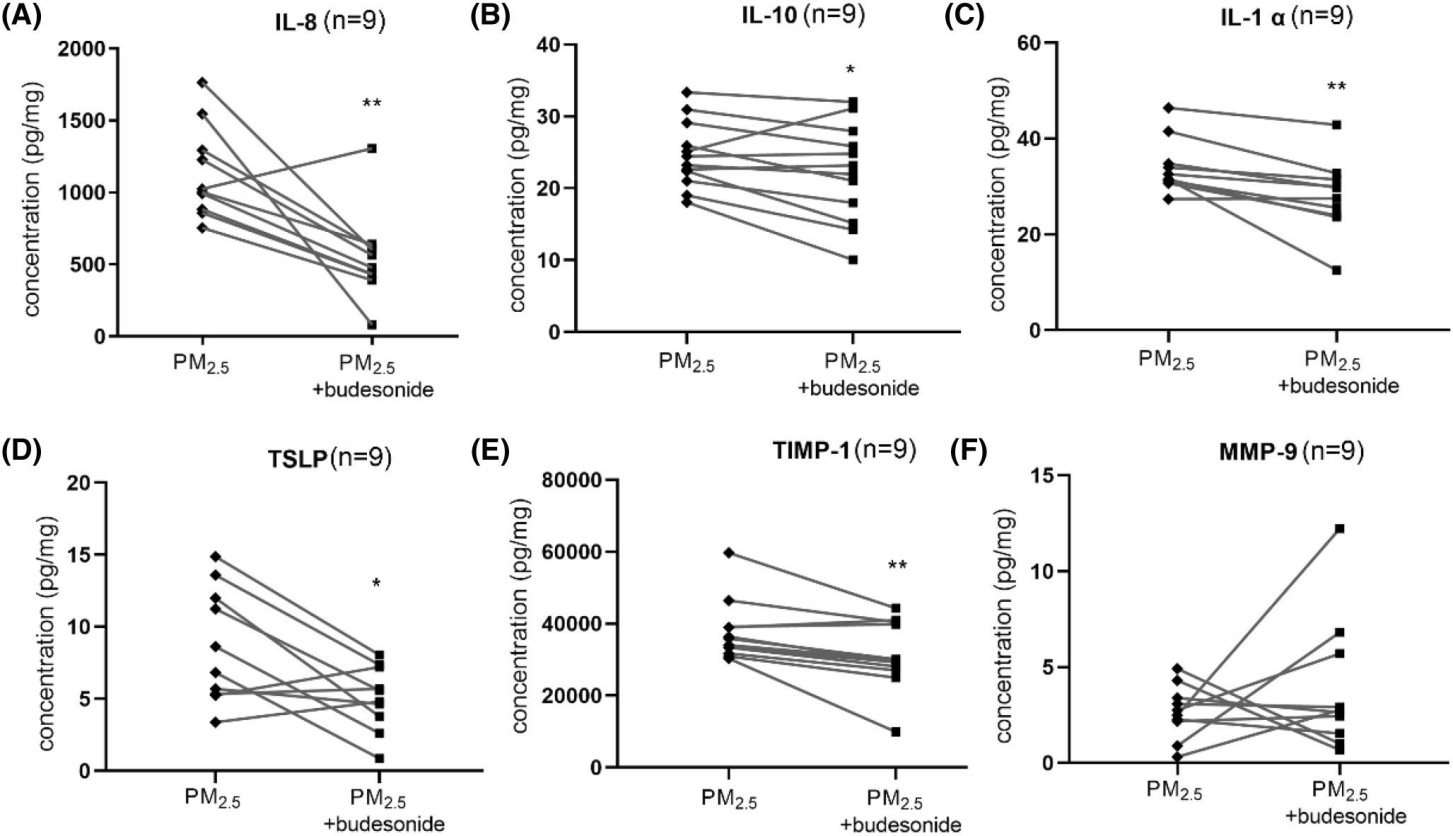
Effects of budesonide on cytokine concentrations in the culture medium. Cells were pretreated with or without budesonide before exposure to 100 μg/ml PM_2.5_, and the concentrations of IL‐8, IL‐*1α*, TIMP‐1, IL‐10, TSLP, and MMP‐9 in the culture medium were evaluated. *p < 0.05. IL, interleukin; MMP, matrix metalloproteinase; PM_2.5_, particular matter 2.5; TIMP, tissue inhibitor of metalloproteinase; TSLP, thymic stromal lymphopoietin

As shown in Figure 6, when the cells were pretreated with budesonide 1 h prior to 100 μg/ml PM_2.5_ exposure, IL‐8, IL‐10, IL‐1*α*, TSLP, and TIMP‐1 levels decreased significantly in the culture medium. MMP‐9 concentrations did not show a clear tendency. For noninflammatory human nasal epithelial cells exposed to PM_2.5_, budesonide pretreatment failed to induce any significant changes (Figure S4).

## 4 DISCUSSION

Epithelial cells are vital in both immune response regulation and host defense, functioning to mediate innate immunity and play important roles in adaptive immunity.^2015^, ^2007^, ^2016^, ^2014^ The impaired barrier function of the nasal epithelial has been shown to be related to type‐2 inflammatory diseases, for example, nasal allergic disease and polyps.^2018^, ^2017^ However, the relationship between the increasing rate of type‐2 inflammation and deteriorating air pollution is still unclear. In this study, we used eos‐CRSwNP epithelial cells as a model of type‐2 inflammatory cells to explore the effects of PM_2.5_ on the barrier function of epithelial cells and its relationship to adaptive immunity. We also investigated a mechanism to repair the damage.

The components of PM_2.5_ vary in different locations, but the key components remain the same.^2014^, ^2015^ Here, we used PM_2.5_ collected in Beijing and processed with a standard procedure used in many studies to ensure that the sample was representative.^2018^, ^2017^

CRSwNP is a heterogeneous disease that can be divided into four inflammatory patterns (Th1/Th2/Th17/Th22).^23^ Similar to allergic rhinitis, eos‐CRSwNP is a type‐2 inflammatory disease^2007^, ^2018^ that is relatively difficult to cure. Therefore, it is reasonable to use epithelial cells from eos‐CRSwNP to establish a relatively homogeneous type‐2 inflammatory model in vitro.

As critical components of the epithelial barrier, TJs restrict the passage of ions, liquids, and larger molecules through pore or leak pathways,^25^ which are commonly reflected by TER and paracellular flux.^17^ However, several studies have shown that TER and paracellular flux are distinct properties of TJs and are not necessarily consistent.^2011^, ^2013^, ^2012^, ^1996^, ^2010^, ^2009^ TER reflects the instantaneous paracellular moving of ions, and the flux is determined by dynamic instability, reflecting the sum of transient breaks for larger molecules.^2012^, ^2009^ Notably, the pore pathway is mainly regulated by the pore‐forming claudin protein family, which not including claudin‐1. In contrast, ZO‐1 and occludin have been shown to regulate the leak pathway^25^ but not the pore pathway. In this study, PM_2.5_ increased paracellular flux but did not change TER, after 72 h of intermittent exposure, implying that PM_2.5_ may have more influence on the leak pathway.

Our previous study showed that PM_2.5_ affected both TER and paracellular flux in noninflammatory ALI‐cultured cells.^12^ The different results may support that eos‐CRSwNP epithelial cells and noninflammatory cells exhibit different intrinsic characteristics, especially for the steroids treatment. Moreover, the TER baseline of eos‐CRSwNP epithelial cells was already significantly lower than that of noninflammatory cells, suggesting that PM_2.5_ may not further decrease TER for the barrier‐defected eos‐CRSwNP epithelial cells.

Budesonide did not cause any significant change in TER but was capable of repairing barrier function according to paracellular flux. Although budesonide has been shown to strengthen TJs, its effects on TER typically do not reflect a treatment that was just applied within 3 days.^2017^, ^2012^ However, because most allergens are proteins with a molecular weight of approximately few kDa,^33^ the reparative effects of budesonide on the leakage of larger molecules may have more clinical significance.

Regarding the influence of PM_2.5_ on TJ proteins, our results were partially consistent with previous studies.^12^ Similar to that in noninflammatory cells, the mRNA levels of TJ proteins were not completely consistent with their protein levels in eos‐CRSwNP epithelial cells. However, in contrast to noninflammatory cells, the mRNA expression levels of claudin‐7, ZO‐1, and ZO‐2 only tended to increase, and this result may be related to the different expression patterns of TJs between noninflammatory and eos‐CRSwNP epithelial cells.^34^

In accordance with previous studies, glucocorticosteroid can increase the expression of ZO‐1, occludin, and claudin‐1.^32^ ^,^ ^35^ In this study, although budesonide did not completely alleviate the effects of PM_2.5_ on paracellular flux and TJ proteins, it partially reversed the trend, in contrast to the results in noninflammatory cells. Thus, steroid treatment may be essential for patients with eos‐CRSwNPs and other nasal type‐2 inflammatory diseases to prevent damage caused by PM_2.5_.

As a bridge between innate and adaptive immunity, cytokines secreted by epithelial cells play important roles in the initiation of inflammation.^36^ IL‐1*α* is a major initiation factor of many inflammatory processes and is referred to as an “alarmin” and a critical danger‐associated molecular pattern.^37^ It was reported that IL‐1*α* can promote the secretion of Th1‐type cytokines, such as IL‐8,^38^ and stimulate the production of pro‐Th2 cytokines, such as IL‐25, IL‐33, and granulocyte macrophage colony‐stimulating factor.^6^ Although rarely reported in the respiratory system, previous studies have shown that PM_2.5_ can promote the secretion of IL‐1*α* in skin and blood.^2017^, ^2017^, ^2019^ In this study, we could not exclude the possibility that IL‐1*α* may be the initiation promotor of IL‐8, and further studies are needed to explore these mechanisms.

IL‐10 is an anti‐inflammatory cytokine that is secreted by respiratory epithelial cells.^2003^, ^1998^ IL‐10 can directly suppress the antigen‐presenting function of antigen‐presenting cells and inhibit the secretion of pro‐inflammatory cytokines, including IL‐1*α*.^44^ Our previous in vivo study showed that IL‐10 is decreased in nasal secretions after PM_2.5_ exposure but increased in the supernatants of peripheral blood mononuclear cells after incubation with PM_2.5_.^45^ In this study, we assumed that elevated levels of IL‐10 may be a self‐protective reaction in epithelial cells, supporting the self‐regulatory function of the epithelial cells without interfering with adaptive immune cells.

Similar to our results observed in noninflammatory cells,^12^ the secretion of TIMP‐1, an inhibitor of MMP‐9, was increased in type‐2 inflammatory cells upon exposure to PM_2.5_. However, we did not observe significant changes for TSLP, potentially because of the higher baseline concentration in type‐2 inflammatory cells.^46^

For the noninflammatory epithelial cells, budesonide could not repaire the barrier disruption caused by PM_2.5_, which is consistent with our previous results.^12^ In this study, we also found budesonide could not alter the cytokine production of noninflammatory cells exposed to PM_2.5_, but significantly reversed the changes of eos‐CRSwNP epithelial cells. Thus, we assume that the influence of corticosteroid maybe varied because of the intrinsic characters of different epithelial cell types and might be related to its effects on cytokines secretion.

However, this study has several limitations. First, the duration of PM_2.5_ exposure in the ALI‐culture model and the number of donors enrolled in this study were limited, which may restrict the clinical significance in this chronic inflammatory model. And this model could not represent completely the in vivo environment. Besides, the mechanisms underlying the damage on barrier integrity caused by PM_2.5_, especially in different inflammatory conditions, are not clear. Furthermore, the mechanisms underlying the reparative function and the heterogenous response of different kinds of NPs to cocicosteroid are not clear, and deserve further study. The difference between noninflammatory nasal epithelial cells and eos‐CRSwNP epithelial cells exposed to PM_2.5_ is interesting and calls for further exploration.

## 5 CONCLUSION

In summary, our study demonstrated that PM_2.5_ could further impair the barrier function of eos‐CRSwNP epithelial cells and increase the permeability of the cells to large molecules. PM_2.5_ could increase the secretion of both type‐1 and type‐2 cytokines from nasal epithelial cells in situ and promote remolding. Finally, although corticosteroid has been already widely used for CRSwNP, our finding that budesonide partially reversed these changes in barrier function and cytokine levels caused by PM_2.5_ implies its role in preventing disease progression in high PM_2.5_ situation.

## CONFLICT OF INTEREST

The authors have no conflict of interest.

## AUTHOR CONTRIBUTIONS

Siyuan Ma and Mu Xian performed the experiments, analyzed the data, and prepared the manuscript; Yang Wang participated in the experiments and data collection; Chengshuo Wang and Luo Zhang were responsible for the overall study design, data analysis, and manuscript revision.

## Supporting information

Supporting Information 1
