# Supplementary material for: Budesonide repairs decreased barrier integrity of eosinophilic nasal polyp epithelial cells caused by PM2.5
Source: Clin Transl Allergy. 2021 Jul 3;11(5):e12019. doi: 10.1002/clt2.12029 (PMC8254582; doi:10.1002/clt2.12029)

**SUPPLEMENTARY TABLES AND FIGURES**

**Table S1.** Primer sequences used for polymerase chain reaction

| Gene | Forward primer | Reverse primer |
| --- | --- | --- |
| EF1α | CTG AAC CAT CCA GGC CAA AT | GCC GTG TGG CAA TCC AAT |
| Claudin-1 | CAG TCA ATG CCA GGT ACG AAT TT | AAG TAG GGC ACC TCC CAG AAG |
| Claudin-4 | TGT ACC AAC TGC CTG GAG GAT | GAC ACC GGC ACT ATC ACC ATA A |
| Claudin-7 | GGG CAT GAA GTG CAC GCG CT | CGG CAA GAC CTG CCA CGA TG |
| Occludin | GAT GAG CAG CCC CCC AAT | GGT GAA GGC ACG TCC TGT GT |
| ZO-1 | ACA GTG CCT AAA GCT ATT CCT GTG A | TCG GGA ATG GCT CCT TGA G |
| ZO-2 | CGG TTA AAT ACC GTG AGG CAA A | GGG AAC CAC TGG GTG TAA TTC A |
| IL-1α | AGATGCCTGAGATACCCAAAACC | CCAAGCACACCCAGTAGTCT |
| IL-8 | ACTGAGAGTGATTGAGAGTGGAC | AACCCTCTGCACCCAGTTTTC |
| IL-10 | GACTTTAAGGGTTACCTGGGTTG | TCACATGCGCCTTGATGTCTG |
| TSLP | ATGTTCGCCATGAAAACTAAGGC | GCGACGCCACAATCCTTGTA |
| TIMP | CTTCTGCAATTCCGACCTCGT | ACGCTGGTATAAGGTGGTCTG |
| MMP-9 | TCTGGAGGTTCGACGTGAAGG | GAACTCACGCGCCAGTAGAA |

*: Sequence direction is “5′-3′” for both primers.

**Figure E1.** (A) The baseline TER of noninflammatory nasal epithelial cells and eos-CRSwNP epithelial cells. (B) After exposure to 100 μg/mL PM_2.5_, the TER of eos-CRSwNP epithelial cells was measured and compared with that in noninflammatory nasal epithelial cells. (C, D) After budesonide pretreatment, the changes in TER caused by 100 μg/mL PM_2.5_ were evaluated in the two cell types. ***P* < 0.01.


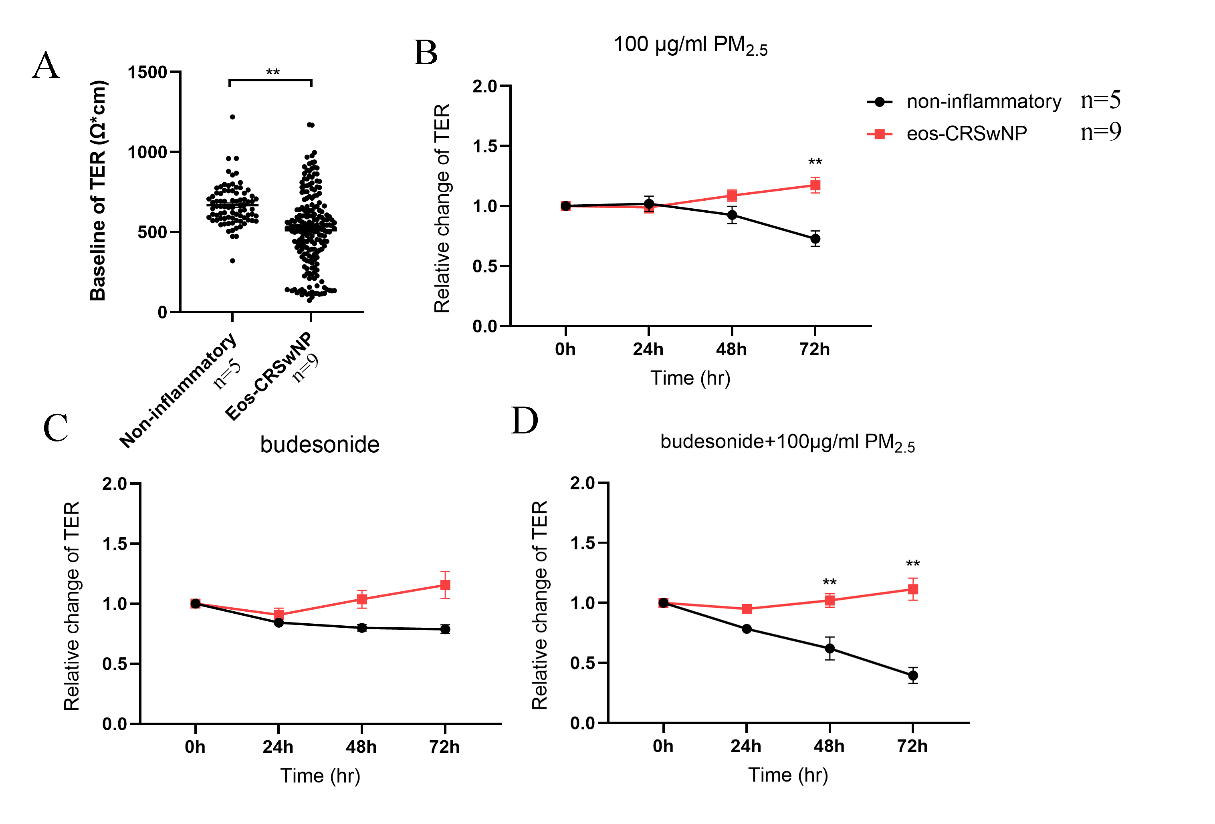


**Figure E2.** Paracellular flux of the ALI-cultured non-inflammatory nasal epithelial cells and eos-CRSwNP epithelial cells with or without PM_2.5_ exposure and budesonide pretreatment.


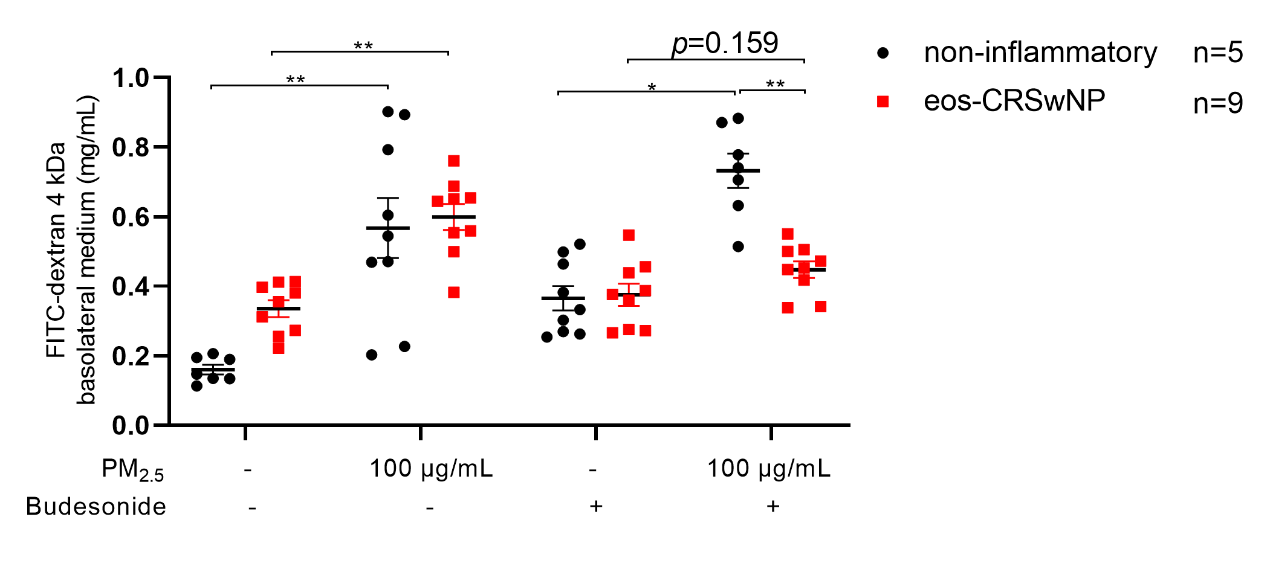


**Figure E3.** Cytotoxicity of PM_2.5_ in eos-CRSwNP epithelial cells. PM_2.5_ was added to the culture medium at concentrations varying from 1 to 1000 μg/mL for 24 h. LDH release in the culture supernatants was measured in triplicate.


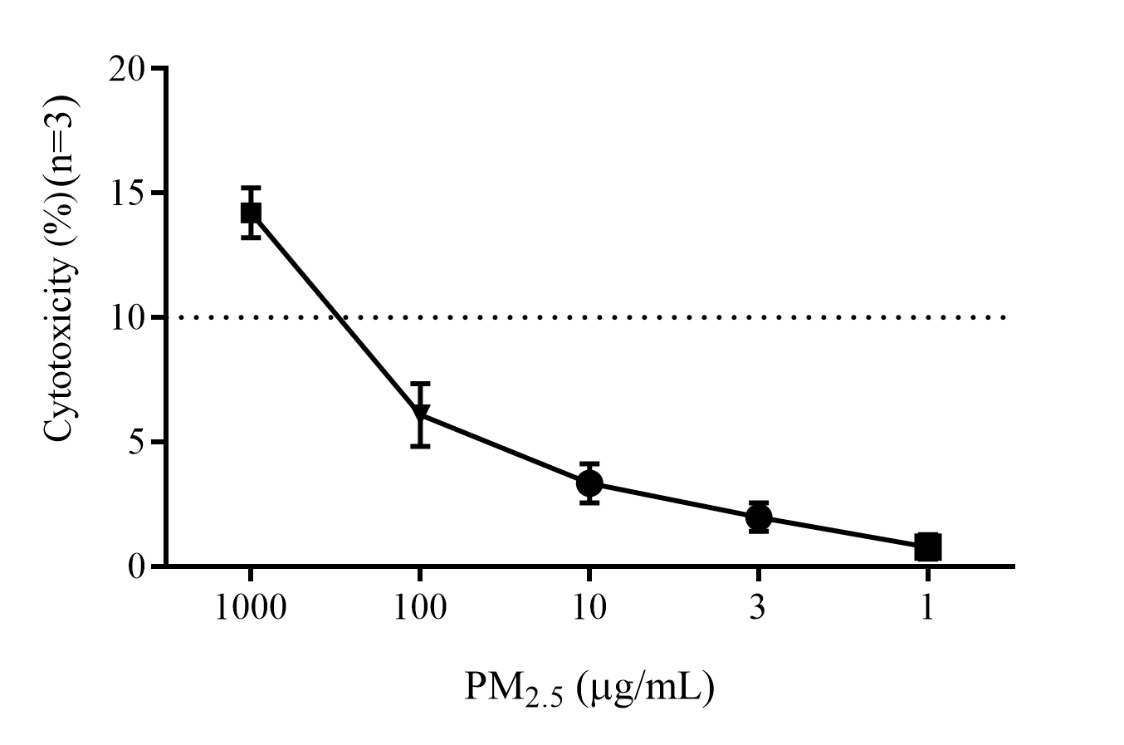


**Figure E4.** Effects of budesonide pretreatment on cytokine concentrations in the culture medium of ALI-cultured non-inflammatory epithelial cells exposed to 100 μg/mL PM_2.5_. IL-8 (A), IL-10 (B), TIMP-1 (C), and MMP-9 (D) were evaluated.


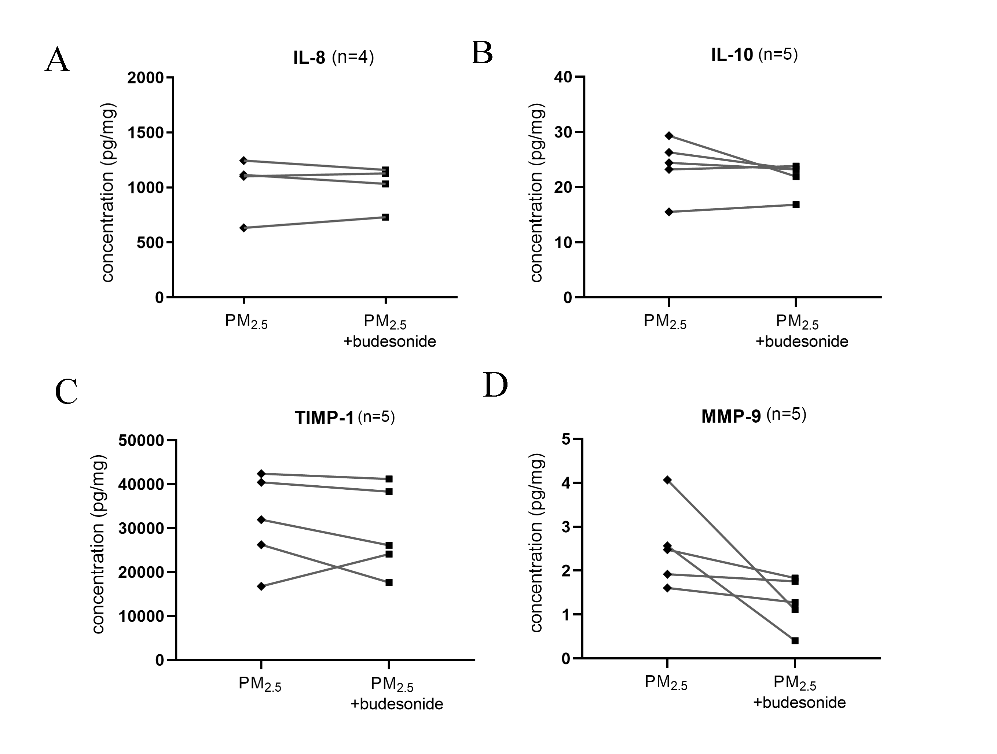

Supplement: Supplementary file 1 — Supporting Information 1 [file CLT2-11-e12019-s001.docx]
